# QoS-Aware Algorithm Based on Task Flow Scheduling in Cloud Computing Environment

**DOI:** 10.3390/s22072632

**Published:** 2022-03-29

**Authors:** Mohamed Ali Rakrouki, Nawaf Alharbe

**Affiliations:** 1 Applied College, Taibah University, Medina 42353, Saudi Arabia; nrharbe@taibahu.edu.sa; 2 Ecole Supérieure des Sciences Economiques et Commerciales de Tunis, University of Tunis, Montfleury 1089, Tunisia; 3 Business Analytics and DEcision Making Lab (BADEM), Tunis Business School, University of Tunis, Bir El Kassaa 2059, Tunisia

**Keywords:** cloud computing, virtual machine placement, scheduling, ARIMA, QoS

## Abstract

This paper deals with the challenging problem of scheduling users’ tasks, while taking into consideration users’ quality of service (QoS) requirements, with the objective of reducing the energy consumption of physical machines. This paper presents a model to analyze the current state of the running tasks according to the results of the QoS prediction assigned by an ARIMA prediction model optimized with Kalman filter. Then, we calculate a scheduling policy with a combined particle swarm optimization (PSO) and gravitational search algorithm (GSA) algorithms according to the QoS status analysis. Experimental results show that the proposed HPSO algorithm reduces resources consumption 16.51% more than the original hybrid algorithm, and the violation of service-level agreement (SLA) is 0.053% less when the optimized prediction model is used.

## 1. Introduction

When users purchase cloud services, they will sign an service-level agreement (SLA) with the service provider. If the service provider violates the SLA, it will cause adverse effects and pay liquidated damages. Therefore, the service provider needs to use various means to ensure the QoS of users when designing cloud systems, to be able to complete on time as agreed. The QoS reflects the satisfaction degree of the various indicators about the service formulated by the user when negotiating with the service provider [1]. Since there are many users’ tasks in the system, it is necessary to select an appropriate virtual machine when assigning tasks, so the system will face the problem of task flow scheduling.

Task flow scheduling refers to the mapping of users’ tasks to cloud computing resources. The cloud provider must take into account the different quality of service (QoS) requirements laid down in the SLA signed with users. Moreover, processing tasks in a cloud computing context raises QoS and energy-related problems, necessitating the use of effective scheduling algorithms [2], and SLA compliance [3] can be achieved at a fair economic cost for end users, while keeping resource energy consumption to a minimum. From the user’s point of view, different users need different service quality. For example, some users are relatively concerned about the response time of the service, and some users care about the service cost. From the provider’s point of view, the cloud provider must first complete the tasks submitted by the users according to their QoS requirements. Completing these tasks requires a series of cloud computing resources, such as CPU, memory, disk, network, and other resources. However, cloud computing data centers are business-oriented, so cloud computing providers need to reduce their costs by reducing the energy consumption of the physical machines.

In this context, this paper considers the problem of task flow scheduling with the objective of minimizing energy consumption of the cloud while taking into account users’ QoS. This challenging problem is a multi-objective optimization NP-hard problem [4] and the research of the solution mainly focuses on heuristic algorithms based on global search in the case of multi-objective constraints [5,6,7,8,9]. First, in order to satisfy user QoS, a scheme is proposed for user task flow scheduling based on QoS prediction results. These results are calculated using an ARIMA (autoregressive integrated moving average) forecasting model based on user QoS [10] in order to predict the future trend of user QoS according to the monitoring data of the system. When the prediction model evaluates that the trend exceeds the safe area, it indicates that the user QoS is in danger. User tasks are scheduled to ensure user QoS. If a suitable scheduling scheme cannot be found, it is necessary to apply for more physical resources to the user. This prediction model is integrated in a hybrid metaheuristic, originally proposed by [11]. This algorithm is based on the particle swarm optimization (PSO) algorithm, and the gravitational effect between particles is added when the particles move in each iteration, so that the search space is larger and the search accuracy is higher. In the introduction of the hybrid algorithm in the original text, there is no plan to reserve the virtual machine in the physical machine before each scheduling. Too many reserved virtual machines result in waste of resources, while too few reserved virtual machines cause great system overhead and energy waste due to the continuous deletion and creation of virtual machines. Therefore, based on the original hybrid algorithm, this paper proposes a virtual machine reservation scheme. The number of virtual machines reserved refers to the idea of the network congestion control algorithm, and the specific virtual machines to be reserved are judged according to the resource utilization. Finally, CloudSim is used to conduct simulation experiments to verify the effectiveness of the improved algorithm.

## 2. Literature Review

According to the needs of different users or providers, the scheduling of cloud computing resources can be divided into the following three categories according to different purposes: reducing energy consumption, improving resource utilization, and economy-oriented.

The energy consumption problem of cloud computing systems is a main research direction of the cloud computing resource scheduling problem, and it is also an important optimization goal responding to the call of green cloud computing [12], reducing the waste of resources, and improving the benefits of cloud computing business models. Ref. [13] studied the problem of energy optimization in virtual cluster environment, and proposed a method to reduce CPU frequency to reduce energy consumption. The main idea is as follows: by monitoring the status of the virtual machine, when its load is less than a certain threshold (cannot affect the user QoS), the processor frequency is actively reduced to reduce energy consumption. However, Ref. [13] does not strictly consider the impact of reducing the processor frequency on task completion, so it may affect the user’s QoS. Ref. [14] considered the problem of minimizing tasks’ completion time and energy consumption. The experimental results show that this method has obvious improvement in energy consumption and completion time compared with the method of [15]. Ref. [16] considered the problem of energy management in green cloud and formulated it as a combinatorial optimization problem with the objective of minimizing energy consumption and optimizing load balancing. In order to solve this problem, Ref. [16] proposed an effective clonal selection resource scheduling algorithm. An improved scheduling algorithm and an optimal energy consumption minimization method were proposed by [17] for dynamic resource allocation problem. The proposed solution outperformed the existing methods by 8%.

In order to improve resource utilization, the current main method is to schedule virtual machines and tasks. Ref. [18] studied the deployment and migration of virtual machines, and proposed an optimized resource management method for total dynamic scheduling time. Refs. [19,20] proposed a dynamic scheduling algorithm for virtual resources, established a constraint satisfaction model for task scheduling and virtual machine scheduling, and then solved the scheduling scheme. Ref. [21] proposed two-level scheduling for system resource utilization: task scheduling and virtual machine scheduling, respectively. Finally, the experiments proved that the effect of optimizing the load can be achieved. In order to optimize the load balancing of virtual machines, a threshold-based task scheduling algorithm was proposed in [22]. Recently, Ref. [23] proposed a novel multi-objective metaheuristic algorithm for minimizing the completion time of tasks and maximizing resource utilization. For predicting CPU utilization, Ref. [24] proposed two predictive methods based on Holt–Winters exponential smoothing (HW) and long short-term memory (LSTM). The experimental results showed that these approaches can reduce CPU slack by over 40% for a variety of CPU workloads. Moreover, as compared to the HW method, the deep-learning-based approach LSTM has been demonstrated to produce more stable predictions. Similarly, Ref. [25] proposed a deep-learning-based method combining conditional restricted Boltzmann machines (CRBMs) and clustering algorithms in order to minimize resource utilization. The experiments showed that the proposed method can improve CPU and memory utilization by 30%, by exploiting resource utilization patterns. Ref. [26] designed a hybrid method by combining cuckoo search (CS) and particle swarm algorithm for minimizing the energy consumption and the makespan of task scheduling. The proposed algorithm was tested on real supercomputing workload traces and the experimental results proved that this algorithm provided more efficient schedules than other well-known methods.

An economic model-oriented scheduling algorithm was proposed by [27,28]. A market-oriented cloud computing architecture, resource allocation, and scheduling algorithm were established. The algorithm model realizes negotiation between users and providers through an SLA resource allocator. Then, Ref. [29] designed a genetic scheduling algorithm to deal with the market supply and demand balance, and the scheduling algorithm is mainly aimed at the underlying resource scheduling. However, this algorithm is only for the scheduling of CPU resources, and does not consider other resource issues of the virtual machine. Ref. [30] established a cloud alliance model composed of multiple providers according to the economic model. When a provider has insufficient resources, it can rent resources from other providers through resource outsourcing, which effectively avoids resource waste and shortage and cannot meet the problem of user service quality. An artificial bee colony algorithm was proposed by [31] in order to ensure QoS during task scheduling and assure scheduling security. The experimental results showed that the proposed algorithm achieved the objective of securing job scheduling while assuring QoS of users. Ref. [32] proposed a new computation-as-a-service (CaaS) cloud platform in order to reduce billing costs. The proposed platform integrated three main methods: specific tasks are reactively assigned to available computing units based on availability and defined time-to-completion limits, Kalman filter algorithm is used to determine the best resource estimation, and the number of units processing workloads is controlled using AIMD (additive increase multiplicative decrease) algorithms. The experiments showed that the proposed platform was able to reduce billing costs by 38% to 500% when compared to the present state of the art in CaaS platforms on Amazon EC2.

Recently, Ref. [33] proposed a whale optimization algorithm for scheduling resources in order to maximize the work completion for meeting users’ QoS. Ref. [34] proposed a method based on a nature-inspired recent algorithm (salp swarm algorithm) for scheduling tasks, in order to satisfy QoS requirements for both users and cloud provider. The proposed algorithm was compared to well-known meta-heuristics and showed better results in terms of makespan, resource utilization, throughput, and average waiting time. Ref. [35] designed a method based on a traditional particle swarm algorithm and QoS-aware task scheduling in order to ensure QoS of users: deadline, scheduling, budget, and reliability. This multi-objective QoS algorithm was tested on a medical cloud platform and the experimental results proved its effectiveness when compared to other known approaches.

A systematic literature review of scheduling approaches in cloud computing environment was presented by [36]. The authors highlight the benefits and drawbacks of existing task scheduling approaches under different cloud environments and for various scheduling objectives. An in-depth investigation and analysis of cloud computing resource scheduling algorithms is provided by [37,38,39].

## 3. Methodology

**Figure 1:**
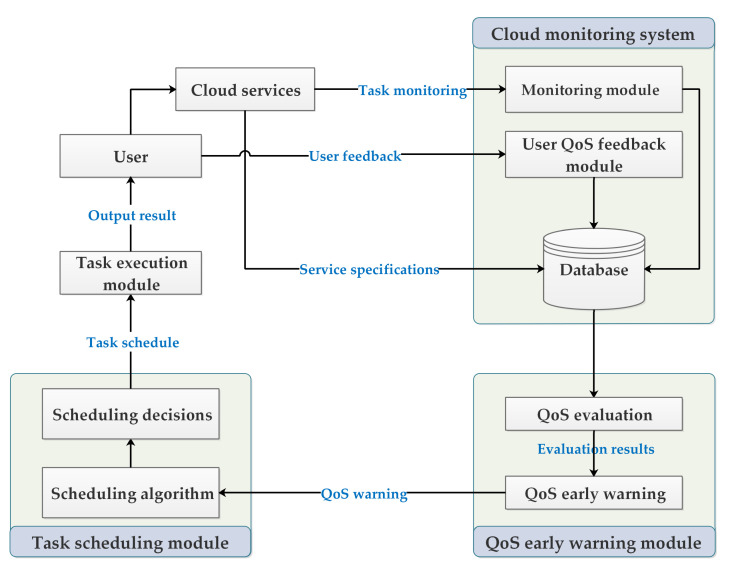
User QoS guarantee overall scheme.

This paper is mainly composed of two modules, user QoS evaluation and early warning module and task flow scheduling module. The cloud service monitoring system is a supporting module and will not be introduced in detail in this paper. From Figure 1, it can be seen that when a user purchases and runs a cloud service, the monitoring module keeps track of the running status of the service and collects data. At the same time, it collects feedback information from the user, and then stores the information in the monitoring database.

The QoS evaluation module is responsible for evaluating the predicted value of QoS according to the monitoring data, service parameter specifications, virtual machine parameter specifications, and other information. Then, this predicted value is transmitted to the QoS early warning module. This module judges whether the user task has QoS danger according to the monitoring data, QoS evaluation value, and QoS classification area, and transfers it to the task scheduling module if there is danger.

The role of the task scheduling module is to schedule the deployment of the task flow in the virtual machine according to the QoS warning, and try to satisfy the QoS of the user through the scheduling of the task flow. The task flow scheduling algorithm is a hybrid algorithm based on PSO and GSA algorithms.

## 4. User QoS Guarantee Model

### 4.1. ARIMA Prediction Model

The ARIMA (autoregressive integrated moving average model) state space model is a time prediction model that combines autoregressive (auto regressive, AR) and moving average (moving average, MA) models. The ARIMA model regards a time-based object sequence as a random sequence. According to the autocorrelation of this sequence, a mathematical model is expected to describe the object sequence [40]. Once it can be described by a mathematical model, then the current and past values of this sequence can be used to predict future values.

1. MA model: The moving average MA model refers to the use of the lag value of the series forecast error to correct the forecast value, and the *n*-order moving average MA refers to the use of the forecast error value of the previous *n* periods.
  (1) $$x_{t}=\theta_{1}\varepsilon_{t-1}+\theta_{2}\varepsilon_{t-2}+...+\theta_{q}\varepsilon_{t-q}+\varepsilon_{t}$$
  where $\theta_{1},\theta_{2},...,\theta_{q}$ are the MA coefficients.
2. AR model: Each term in the expression represents the lag value of the unconditional residual prediction, and the AR model is derived from the first *n* points of the series to the subsequent data.
  (2) $$x_{t}=\phi_{1}x_{t-1}+\phi_{2}x_{t-2}+...+\phi_{p}x_{t-p}+\varepsilon_{t}$$
  where $\phi_{1},\phi_{2},...,\phi_{p}$ are the AR coefficients.
3. Single-integer order *I*: The time series is not necessarily stationary, and the noise in the sequence can be eliminated by sub-difference of the time series in order to obtain a stationary sequence. This stationary series can then be processed using the ARMA model.
4. ARMA model: ARMA is an autoregressive moving average model formed by combining AR and MA models. If a sequence of objects is stable, then the autocorrelation function and partial correlation function of this sequence can be calculated. If both functions are tailed, then the sequence conforms to the ARMA(p,q) model. However, before using ARMA, nonstationary data must be transformed into stationary series using difference methods. Therefore, the ARIMA model is proposed.
5. ARIMA model: It is an improved model of ARMA. It adds differential processing of stationary data and provides a way to integrate AR and MA models. In the ARIMA(p,d,q) model, AR represents autoregression, *p* represents autoregressive term, MA represents moving average, *q* represents moving average term, and *d* is the number of differences in the stationary period of the object series. The ARIMA model has one more differential process than the ARMA model, as follows:
  (3) $${\hat{x}}_{t}=\phi_{1}x_{t-1}^{\prime }+...+\phi_{p}x_{t-p}^{\prime }+\theta_{1}\varepsilon_{t-1}+...+\theta_{q}e_{t-q}+\varepsilon_{t}$$
  where $x_{t}^{\prime }$ represents the difference expression *d* of the object sequence. If d=0, it is recorded as ARIMA(p,0,q), then ARIMA is equal to the ARMA model. Equation (4) gives the calculation of the predicted value at the next time point.
  (4) $${\hat{x}}_{t+1}=\phi_{1}x_{t}^{\prime }+\ldots +\phi_{p}x_{t-p}^{\prime }+\theta_{1}\varepsilon_{t}+\ldots +\theta_{q}\varepsilon_{t-q}+\varepsilon_{t}$$
  If p,d, and *q* can be determined in the ARIMA(p,d,q) model, then the future forecast value can be easily calculated by Equation (4) and monitoring data.

### 4.2. Kalman Filter

In the context of state space models, Kalman filter [41] is an algorithm for estimating unknown variables based on observations collected over time. This algorithm has proven to be effective in a variety of applications. Kalman filters are simple in design and need little computation time. However, because the Kalman filter can be applied to any state space model, it is used in this paper to fit the ARIMA model presented previously. As a result, combining Kalman filter with ARIMA could result in more accurate predictions thanks to optimal estimations [42]. A detailed description of the algorithm is presented in [43].

**Figure 2:**
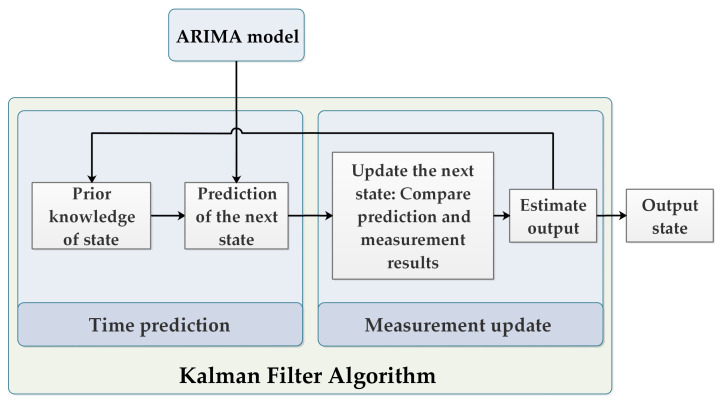
Kalman filter algorithm.

The Kalman filter algorithm consists of two main steps. The first step is to make a state prediction. The filter is then given input in the form of measurements, which can be noisy and imperfect. As a result, the filter is divided into two components, time prediction and measurement update, as illustrated in Figure 2.

### 4.3. User QoS Warning Decision

In order to accurately describe the QoS status of users, the cloud must have a real-time monitoring system that can monitor the QoS status of users. This system is responsible for monitoring various parameter data of virtual machines, response time of user tasks, resource consumption, and resource utilization. This basic monitoring information is not described in detail in this paper.

In order to formulate an early warning system for QoS, it will be necessary to quantify user QoS. The following are explanations of several terms and phenomena of QoS that will be used:

1. QoS status: In a monitoring time period, according to the QoS monitoring data, the current status of the user QoS is divided into areas: normal area, early warning area, dangerous area, and failure area.
2. QoS degradation: In two monitoring time periods, QoS value is decreasing. In other words, the QoS state is deteriorating.
3. QoS rises: In two monitoring time periods, QoS monitoring value develops in a good direction, indicating that the QoS is in a rising stage at this time.
4. Minimum QoS standard for users: This is the minimum QoS standard agreed upon by both parties when the user and the provider sign an SLA or contract. The provider must complete requests submitted by the user with quality and quantity within the agreed time. For tasks, the minimum QoS criteria are mainly the response time and the successful completion of the task.
5. Service failure: The task submitted by the user was not completed on time, or was not completed successfully.

**Figure 3:**
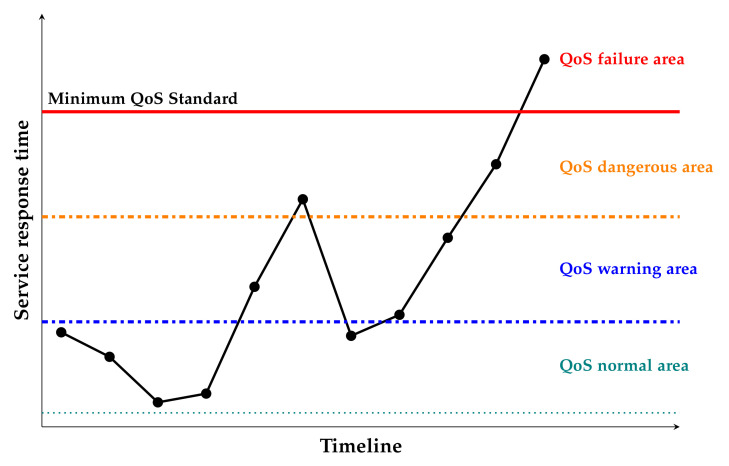
User QoS state diagram.

We need to use a QoS variation value ΔQ to measure the current state of QoS.

(5) $$\Delta Q=\min_{i}\left(\Delta q_{i}\right)$$

where $q_{i}$ represents each QoS index; the larger the value of ΔQ, the farther the current value is from the minimum standard, and the better the user QoS state. $\Delta q_{i}$ is calculated by Equation (6).

(6) $$\Delta q_{i}=\left\{\begin{array}{c} \begin{array}{cccc} |q_{i}^{p}- & q_{i}^{f}| & \; & ;\mathrm{if}\;\mathrm{within}\;\mathrm{the}\;\mathrm{scope}\;\mathrm{of}\;\mathrm{user}\;\mathrm{standards}\\ 0 & \; & & ;\mathrm{if}\;\mathrm{exceeds}\;\mathrm{user}-\mathrm{set}\;\mathrm{standards} \end{array} \end{array}\right.$$

where $q_{i}^{p}$ is the QoS value calculated at a certain monitoring time point, and $q_{i}^{f}$ is the corresponding minimum user QoS standard. According to the floating range of QoS, the current user QoS status is divided into four categories (see Figure 3):

1. QoS normal area: The user’s task can obtain the response of the server in a very timely manner, and the user is satisfied with the current service.
2. QoS early warning area: The user’s task can obtain timely response from the server, but the QoS response time of the task is slightly higher than that of the normal area, indicating that the user’s QoS is in a relatively dangerous area. At this time, the prediction data of the prediction module are needed to judge the trend of user QoS. If the QoS trend has a serious downward trend, then the scheduling center needs to schedule the user’s tasks to ensure that the user’s QoS is in the normal area or develops toward the normal area.
3. QoS dangerous area: The response time of the user’s task is close to the bottom line set by the user. If the prediction data indicate that the QoS is not on the rise, then the scheduling center needs to schedule the user’s task and allocate resources to it with high priority.
4. QoS failure area: The user’s task cannot be responded to in time by the service, and then it will not be completed on time. Thus, the user’s loss should be calculated and compensated according to the contract. Therefore, it must be ensured that the user’s QoS does not reach the failure area.

According to the prediction result of user QoS and the current monitoring value, the QoS change trend of the user can be calculated, and the QoS change trend can be divided into four states according to the value range of the change trend:

1. QoS rises: QoS status changes to a good direction;
2. Slow QoS degradation: QoS status deteriorates, speed is low;
3. QoS medium-speed degradation: QoS state deteriorates, the speed is medium;
4. QoS high-speed degradation: QoS state deteriorates, speed is high.

Then, by integrating QoS status and QoS trend, we can obtain the QoS early warning decision, as shown in Table 1.

### 4.4. QoS Guarantee Processing Flow

**Figure 4:**
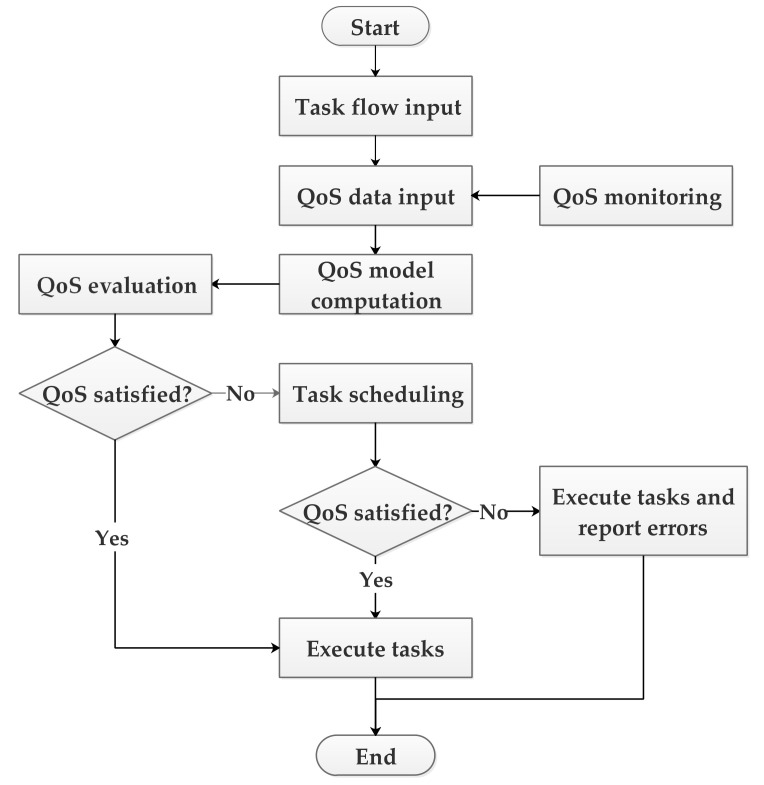
Task execution process based on user QoS guarantee.

Figure 4 shows the task scheduling process based on user QoS guarantee. First, the system obtains a user’s task flow input according to the user’s task request and user QoS. The submitted task run in the virtual machine in the cloud system. During operation, the monitoring system can obtain various monitoring information about the task and the virtual machine, and calculate the current status of the user’s QoS. Then, the current QoS state is evaluated. If the evaluation result complies with the QoS evaluation model standard, the task will continue to be executed until the end. If the evaluation result shows that the user’s QoS is in a dangerous state or exceeds the normal range of the user’s QoS, then in the next task scheduling, a scheduling scheme that is beneficial to the user’s QoS will be calculated and scheduled. The monitoring of QoS status of tasks continues after scheduling until the task ends. If the evaluation results after scheduling still cannot meet the user’s QoS, the system will issue an alarm.

## 5. Task Flow Scheduling Algorithm

The task flow scheduling in cloud computing is a discrete problem, but since the particle swarm is a continuous model, it is necessary to mathematically model the particles to correspond to the task flow scheduling scheme, so that the solution of PSO algorithm corresponds to the task flow scheduling. In the following is an introduction to algorithm modeling.

**Figure 5:**
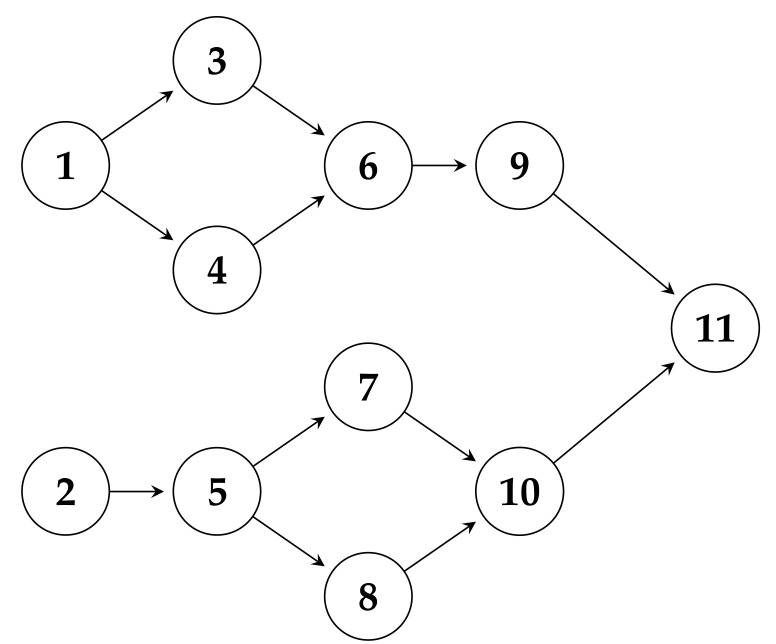
Task flow DAG topology.

The task flow in cloud computing can be regarded as a topological sequence, such as in Figure 5, which is a directed acyclic graph (DAG). Each node in the graph represents a cloud task, and each edge represents the execution order of the tasks. Now, map the tasks to the particles in the PSO algorithm: each particle in the algorithm is a solution and represents a scheduling scheme. The dimension number of a particle corresponds to the number of the tasks, and the value of the space dimension indicates the number of the virtual machine to which the task is deployed. The dimension of the particle is d=n, where *n* represents the number of tasks to be scheduled. Particles are constantly moving in space to search for a best scheduling scheme of the task. The quality of the scheduling scheme is judged through a fitness function. Because each particle represents a solution, the larger the number of particles, the larger the search space, but too much search will bring additional overheads. Generally, the number of particles will be greater than the number of tasks.

According to the task flow topology in Figure 5, a scheduling encoding represented by a one-dimensional string can be derived.

**Figure 6:**
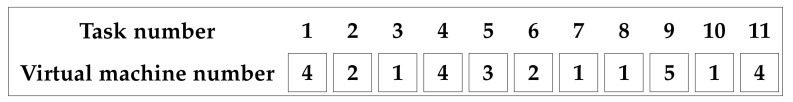
Assignment of tasks to virtual machines.

The task corresponds to the assigned virtual machine number. It can be seen from Figure 6 that each task number corresponds to an assigned virtual machine number, which can be represented as a slice of a particle in one dimension in a certain number of iterations. Figure 6 indicates which virtual machine tasks 1 to 11 are allocated to. This allocation method can be represented by an 11-dimensional particle, and the number on the 11th dimension of the particle represents the virtual machine number. The solution corresponding to the task assignment strategy can be represented by a particle in the particle swarm. The dimension of the particle corresponds to the task number, and the number of the spatial position of the particle corresponds to the assigned virtual machine.

When scheduling tasks, in addition to satisfying the user’s QoS, it is also necessary to reduce the energy consumption of physical machines and their total running time. The main consumption of a task on the system is its running time multiplied by the resources occupied by it, and the amount of data transmission performed by the task on the virtual machine. The running time can be estimated through historical data, and the amount of data transfer is an agreement with the user. Therefore, the total consumption $fit(T_{i})$ of the task *i* can be obtained.

(7) $$fit\left(T_{i}\right)=\omega_{1}^{i}P_{i}+\frac{\omega_{2}^{i}S_{i}}{L}$$

where ω is a correction parameter and $P_{i}$ is the estimated processing time of the task *i*. The first call can take a similar time according to the historical database, and the time is predicted by Equation (4) when the task is migrated; $S_{i}$ is the size of the data that the task *i* needs to transfer, including the size of the task processing data and the number of transfers of the migration task; *L* is the I/O speed of the data center, and the scheduling objective is to minimize the total consumption function fit(T).

### 5.1. Introduction to Hybrid Particle Swarm Optimization—HPSO

As a metaheuristic algorithm, PSO has excellent search ability for near-optimal solutions. However, there are also some shortcomings. PSO can easily fall into local optima, and the solution result is dependent on the velocity parameters.

The gravity search algorithm (GSA) was proposed in 2009 [44]. This algorithm adjusts the position of the solution space by transforming an attribute into mass between objects which are then gravitationally affected by each other, and it finally obtains the best solution through multiple iterations.

The following will introduce an algorithm that combines GSA and PSO algorithms in order to realize task scheduling based on user QoS. By calculating the gravitational force between particles, the algorithm expands the search range of the particle solution space and improves the search accuracy of the particle, which makes up for the deficiency of PSO.

#### 5.1.1. Introduction to Particle Swarm Optimization

The PSO algorithm process is as follows. Each particle *i* has a *D*-dimensional position vector $x_{i}=\left(x_{i1},x_{i2},...,x_{iD}\right)$ and a velocity vector $v_{i}=\left(v_{i1},v_{i2},...,v_{iD}\right)$. The algorithm iteratively calculates the movement of particles. When particle *i* searches the solution space in each iteration, the best position in the moving process is judged by a fitness function and saved as $p_{i}=\left(p_{i1},p_{i2},...,p_{iD}\right)$. In each iteration, the particle’s current velocity consists of three parts: particle inertia, effect of the particle’s best position on the current one, and effect of the swarm’s best position $p_{g}=\left(p_{g1},p_{g2},...,p_{gD}\right)$ on the particle itself. The particle velocity can be calculated according to Equation (9), where $c_{1}$ and $c_{2}$ are positive numbers, called acceleration factors, $r_{1},r_{2}\in \left[0,1\right]$, which can be adjusted and interfered according to requirements, *d* is a dimension in *D* dimension, and ω is the inertia weighting factor.

The new position $x_{id}^{t+1}$ of the particle is calculated from the current position $x_{id}^{t}$ and velocity $v_{id}^{t+1}$, as in Equation (8).

(8) $$x_{id}^{t+1}=x_{id}^{t}+v_{id}^{t+1}$$

(9) $$v_{id}^{t+1}=\omega v_{id}^{t}+c_{1}r_{1}\left(p_{id}^{t}-x_{id}^{t}\right)+c_{2}r_{2}\left(p_{gd}^{t}-x_{id}^{t}\right)$$

The first part $\omega v_{id}^{t}$ in Equation (9) is the inertial velocity of the particle. The larger the inertial velocity, the stronger the global search ability of the velocity, but it will affect the local search accuracy of the particle, and vice versa. The second part $c_{1}r_{1}\left(p_{id}^{t}-x_{id}^{t}\right)$ is the thinking of the particle itself, that is, the influence of the best position saved by the particle on the current speed, and the effect is affected by $\left(c_{1},r_{1}\right)$ adjustments. This form increases the global search ability of particles and avoids local optima. The last part $c_{2}r_{2}\left(p_{gd}^{t}-x_{id}^{t}\right)$ is the influence of the particle by the best position of other particles. This form shows that the particles influence each other, which is a cooperative embodiment of multi-objective optimization. This part is also subject to parameter adjustments.

#### 5.1.2. Introduction to Gravitational Search Algorithm

The gravitational search algorithm (GSA) was proposed by [45]. The algorithm models the problem as multiple particles, each of which is affected by the gravitational force of other particles. This force provides particle acceleration, enabling them to move in the solution space. The mass of a particle is affected by a moderate value, and the larger the moderate value, the greater the mass and the greater the gravitational force on other particles. Therefore, the particles farther away from the optimal solution position have low mass and are easily affected by other particles in the optimal solution position. They move to them and have good local search ability. Gravitational search is not affected by environmental factors, but shares optimized information between particles through gravitational actions between particles.

The particle swarm is represented by Equation (10).

(10) $$X_{i}=x_{i}^{1}+x_{i}^{2}+\cdot +x_{i}^{d}+\cdot +x_{i}^{n};i=\left(1,2,...,N\right)$$

where $x_{i}^{d}$ represents the position of the particle in the *d*^th^ dimension.

The calculation of gravity between particles refers to the formula of universal gravitation as follows.

(11) $$F_{ij}^{d}\left(t\right)=G\left(t\right)\frac{M_{i}\left(t\right)\cdot M_{j}\left(t\right)}{R_{ij}\left(t\right)}\left(x_{j}^{d}\left(t\right)-x_{i}^{d}\left(t\right)\right)$$

(12) $$a_{i}^{d}=\frac{F_{i}^{d}}{M(t)}$$

Equation (11) is a modification of the gravitational formula, where $F_{ij}^{d}\left(t\right)$ represents the gravitational force between particles at time *t*. It can be seen that the distance inside is not squared, because according to [44], it is shown through experiments that the distance is better than the squared distance. The distance between two particles can be represented by the Euclidean distance, as in Equation (13).

(13) $$R_{ij}\left(t\right)={\left\|X_{i}\left(t\right),X_{j}\left(t\right)\right\|}_{Z}$$

Particle *i* will be attracted by other particles, and the gravitational force $F_{i}^{d}\left(t\right)$ of the particle *i* is calculated by Equation (14).

(14) $$F_{i}^{d}\left(t\right)=\sum_{j\in k-best,j\neq i}F_{ij}^{d}\left(t\right)$$

where k−best means the *k* best particles at a certain time. These particles will have a gravitational pull on other random particles, causing a new round of positional changes. The new particle velocity $v_{i}^{d}\left(t+1\right)$ is calculated from the current velocity plus acceleration $a_{i}^{d}\left(t+1\right)$. The acceleration is calculated from gravity, and the new particle position $x_{i}^{d}\left(t+1\right)$ is calculated from the current position plus the displacement per unit time.

(15) $$v_{i}^{d}\left(t+1\right)=rand_{i}\cdot v_{i}^{d}\left(t\right)+a_{i}^{d}\left(t+1\right)$$

(16) $$x_{i}^{d}\left(t+1\right)=x_{i}^{d}\left(t\right)+v_{i}^{d}\left(t+1\right)$$

Then, the particle with the best position best(t) and the particle with the worst position worst(t) are solved by a minimum optimization algorithm.

(17) $$best\left(t\right)=\min fit_{j}\left(t\right);\forall j\in \left\{1,2,...,N\right\}$$

(18) $$worst\left(t\right)=\max fit_{j}\left(t\right);\forall j\in \left\{1,2,...,N\right\}$$

The mass of the particle is obtained by solving the corresponding fitness value of the position of the particle. At time *t*, the mass of the particle is represented by $M_{i}\left(t\right)$. The mass fitness value in this paper is given by Equation (7). The larger the mass of the particle, the better the local optimization of the particle, the closer to the optimal solution, and the greater the gravitational force on other particles. The mass $M_{i}\left(t\right)$ is calculated according to Equations (19) and (20).

(19) $$M_{i}\left(t\right)=\frac{m_{i}\left(t\right)}{\sum_{j=1}^{N}m_{j}\left(t\right)}$$

(20) $$m_{i}\left(t\right)=\frac{fit_{i}\left(t\right)-worst\left(t\right)}{best_{i}\left(t\right)-worst\left(t\right)}$$

The gravitational algorithm is gradually stable in the iterative process, so the gravitational constant G(t) is gradually reduced. Equation (21) gives the solution for the gravitational constant G(t).

(21) $$G\left(t\right)=G_{o}\cdot e^{-a(1/T)}$$

The initial value $G_{o}$ is set to 100, the initial value *a* is set to 20, and the parameter *T* is the number of iterations of the algorithm.

The advantage of the gravitational algorithm is that the positions of some particles are very optimized. In order to improve the convergence speed of the algorithm, some well-optimized particles are selected in each round, and these particles will attract other particles. The selected number directly affects the performance of the algorithm. Therefore, at the beginning of the iteration, this paper selects all particles as the optimized particles which have a gravitational effect on other particles. As the number of iterations *i* increases, the number of optimized particles decreases linearly [44], so in the end, only 2% of the particles will be selected as optimized particles, and the selected number is given by Equation (22).

(22) $$k\_best\_size=p\cdot \left(i-\frac{0.02-T}{1-T}\left(i-1\right)\right)$$

### 5.2. A Hybrid Algorithm Based on PSO and GSA

The algorithm proposed by [11] combines PSO and GSA algorithms. The algorithm uses PSO to complete the global search of particles, and then uses GSA to complete the local search task of particles. The two algorithms have a clear division of labor, and can well solve the problem that global search and local search cannot take into account, and experiments show that the hybrid algorithm has a fast convergence speed and can avoid local optimal solutions well. However, Ref. [11] does not provide a virtual machine reservation scheme for the scheduling period. The following is the scheme proposed in this paper.

When the task flow starts scheduling, there are two tables; one is the virtual machine table, and the other is the task table to be allocated. First, according to the historical data of cloud computing, the virtual machine that needs to be reserved for the next scheduled task needs to be selected from the virtual machine table. This is because retaining a virtual machine saves more resources and time than deleting and then creating a new virtual machine. However, retaining too many virtual machines may cause a waste of resources, and the reserved virtual machine model may not be consistent with the next task scheduling. If it matches completely, it will inevitably cause a large number of resource fragments, reduce resource utilization, and cause waste. Therefore, a strategy is required to decide how many virtual machines to reserve and which ones to reserve.

Firstly, we need to select the number of virtual machines. The number should not be too large or too small. This paper is inspired by the network congestion control algorithm. Both algorithm scenarios need to find a maximum value in a dynamic model, and this value is still changing. Both scenarios can adjust their own policies according to the feedback information of the other party. The congestion algorithm is judged by the maximum value and the transmission of data packets, and the virtual machine reservation can be judged by its operation. However, the fluctuation of cloud computing in general will not be as large as the fluctuation of network transmission, so the specific strategy will be different. Therefore, through the above analysis, there are three algorithm strategies for retaining the number of virtual machines: in recovery phase, the number is increased by multiplication; in the hold stage, the number is increased in small steps; in the fallback phase, the number is multiplicatively decremented.

**Figure 7:**
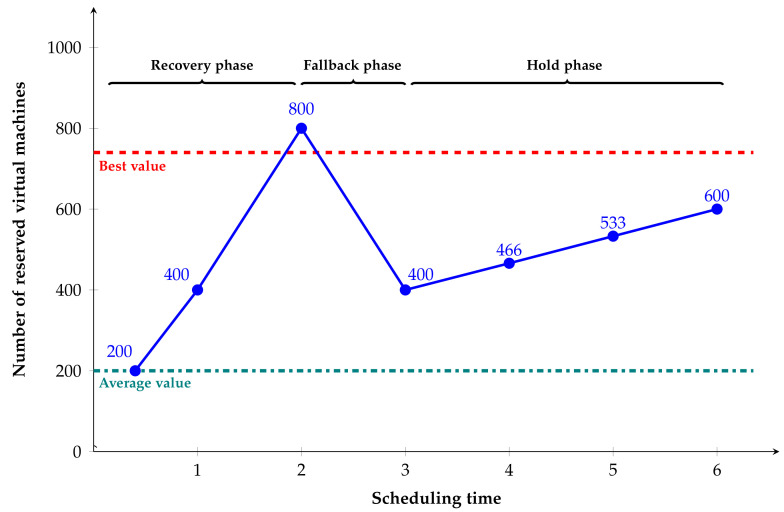
Schematic diagram of virtual machine reservation policy.

According to the historical data, the percentage of the number of virtual machines and the optimal percentage value for maintaining a good running state in each scheduling round are calculated, and then the average and optimal percentage values are calculated according to the current number of virtual machines. The initial number is set to the average number value. For better understanding, it is assumed that the calculated average value is 200, the optimal value is 733, the multiplication parameter in the recovery stage is set to 2, the multiplication parameter in the fallback stage is also 2, and the addition step size in the holding stage is 66. The number of virtual machines retention policy is shown in Figure 7.

After determining the number of reserved virtual machines, we need to determine which virtual machines to reserve. This paper adopts the judgment based on machine load, and allocates the virtual machine with high load.

### 5.3. Algorithm Process

During the operation of the cloud computing system, the provider provides services to cloud users in an on-demand mode, and in order to collect and analyze data, it is necessary to monitor the entire system and increase the number of virtual machines in a timely manner to meet user requests. During the scheduling process, the scheduling center will estimate the task based on historical data. If the existing virtual machine cannot meet the user QoS, the user task will be assigned to a virtual machine with more powerful performance. The scheduling center schedules tasks according to the user’s to-be-assigned task list and cloud resources in hand. The specific process of the proposed method is proposed in Algorithm 1.
**Algorithm 1** HPSO algorithm1:Retain virtual machines according to the reservation scheme;2:Initialize the number of particles according to the number of tasks;3:Set the initial speed of particle swarm algorithm in Equation (8);4:**while** not reaching the maximum number of iterations **do**5:   Calculate particles velocity by Equation (8);6:   Calculate the positions of particles by formula Equation (9);7:   Calculate the mass of particles by Equations (19) and (20);8:   Find the $k_{best}$ particles by Equation (17);9:   Select particles with poor positions by Equation (18);10:   Calculate the gravitational force of particles by Equation (11);11:   Update particles accelerations by Equation (12);12:   Update particles positions by Equation (16);13:   Update the gravitational parameters by Equation (21);14:   Find out the particle solution set with the best predicted value by Equation (7);15:**end while**16:**return** The best solution

It can be seen from the above algorithm that in each round of iteration, the particles are first moved by the PSO algorithm, and then some particles are locally searched by the GSA. In this way, the two algorithms complement each other, expand the search space, and increase search accuracy.

## 6. Experimental Results

The simulation experiments in this paper use CloudSim [46]. The pricing method of virtual machines and the configuration parameters of virtual machines in the experiments refer to Amazon EC2 standards, and are set as small virtual machines, medium virtual machines, large virtual machines, and extra large virtual machines, according to the specifications shown in Table 2. The input tasks is a task flow DAG, and the attributes of each task are the same. In CloudSim, the file input size and output size are both set to 1 GB.

For the parameters of the PSO algorithm, refer to the experimental settings of [47]. In order to expand the search space, the number of particles is set to be twice the number of tasks, and the initial speed of particles in PSO is a random number. The maximum number of iterations is set to 400, and the particle’s inertia value is 0.9. The initial velocity of particles in GSA algorithm is set to 0. The scheduling time of the entire scheduling algorithm cannot be greater than 100 ms, each experiment is run 20 times, and the final data are averaged.

An important indicator in the experiments refers to Equation (7), where $\omega_{1}$ and $\omega_{2}$ are both 0.5. The first is the consumption of the test task running (processing) on the virtual machine, and the second is the data transmission consumption of the task.

**Figure 8:**
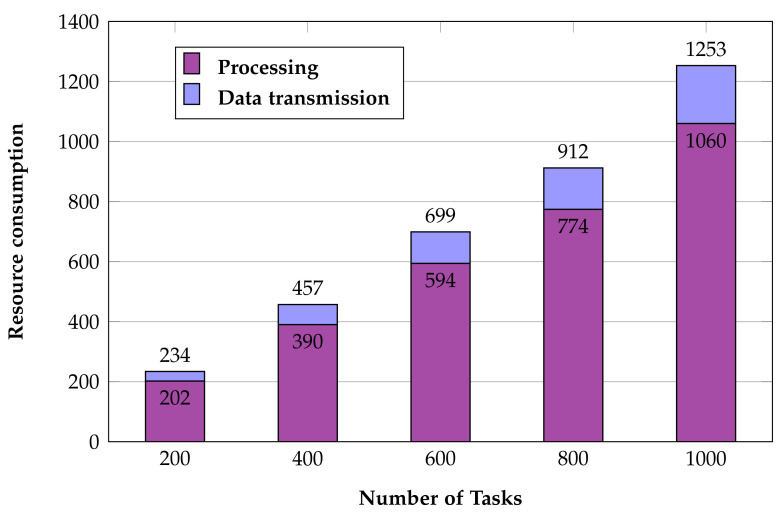
The consumption costs of executing tasks.

Because the time consumption of the ordinate in Figure 8 is weighted in the calculation process, there is no unit added here. The lower the cost in the figure, the better the performance of the algorithm. It can be seen from the experimental results that the main consumption is the processing of the task, mainly because the task occupies the processor core for calculation. We remark that the running consumption occupies more than 80% of the total consumption. When scheduling, it is necessary to reasonably allocate tasks according to their consumption. Because virtual machines come in different sizes, more resource-intensive tasks will tend to be allocated to larger virtual machines.

### 6.1. Performance Analysis

In order to assess the performance of our proposed HPSO algorithm, it is compared to the following algorithms:

1. Greedy algorithm: This algorithm selects the cheapest virtual machine that can meet the deadline of the task to deploy the task, and uses historical data to roughly estimate the running time and end time of the task. The disadvantage of the greedy algorithm is that it does not consider the total consumption of tasks on the virtual machine, and it is easy to fall into local optimum.
2. PSO algorithm [47]: The parameter settings are consistent with those in the HPSO algorithm.
3. GSA algorithm: The parameter settings are consistent with the GSA in HPSO.
4. P-G algorithm [44]: This algorithm makes the solution space search larger and more accurate by combining PSO and GSA.

The performance index of the algorithm refers to Equation (7), which mainly includes the running cost of the task and the cost of data transmission. The goal of the algorithm is to keep this cost as low as possible.

**Figure 9:**
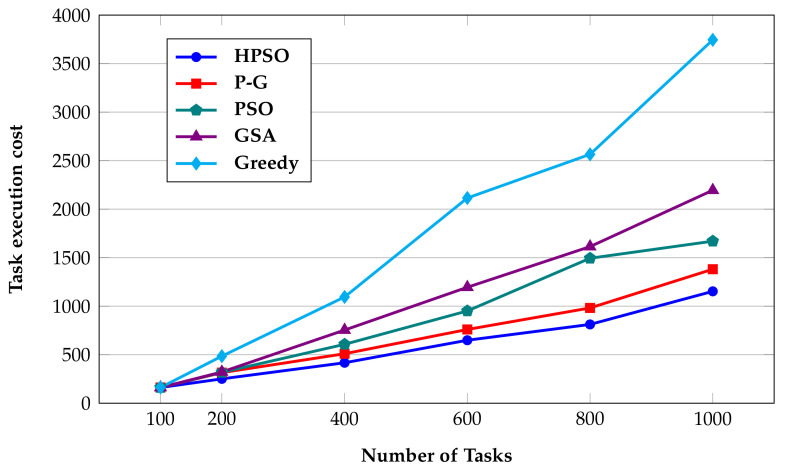
Consumption comparison chart.

As can be seen from Figure 9, the greedy algorithm increases the task execution cost significantly when the number of tasks increases, because it is prone to generate local optimal solutions, which is not obvious when the number of tasks is small. However, when the number of tasks increases, the shortcomings become obvious. The GSA algorithm has significant improvement over the greedy algorithm, but it is slightly worse than P-G and PSO algorithms. This is because the global search ability of the gravitational search algorithm is relatively poor, and the particles can form a good local search ability due to the gravitational interaction between each other. However, gravity is a search technique that keeps particles confined to nearby spaces, rather than spreading out across other spaces. Although the PSO algorithm has limited global search capabilities, a higher initial velocity can be given to the particles to optimize its global search ability. Therefore, the performance of PSO is slightly better than GSA.

The P-G algorithm uses the global search ability of the PSO algorithm, so that particles can first generate a better global position in each iteration, and then use the GSA algorithm to fine-tune the particles. In this way, the particle search is divided into two steps, so it has a better search effect. In addition, it has better convergence in the case of increasing number of tasks.

The improved algorithm HPSO proposed in this paper increases the virtual machine reservation scheme. In each iteration of task scheduling, an algorithmic decision is made on the reserved quantity and quality of virtual machines. Therefore, it can effectively reduce the waste of resources and increase their utilization rate. Compared with the original algorithm P-G, when the number of tasks is low, there is not much change, and when the number of tasks reaches 1000, the execution cost is reduced by 16.51%.

### 6.2. QoS Experimental Results

**Figure 10:**
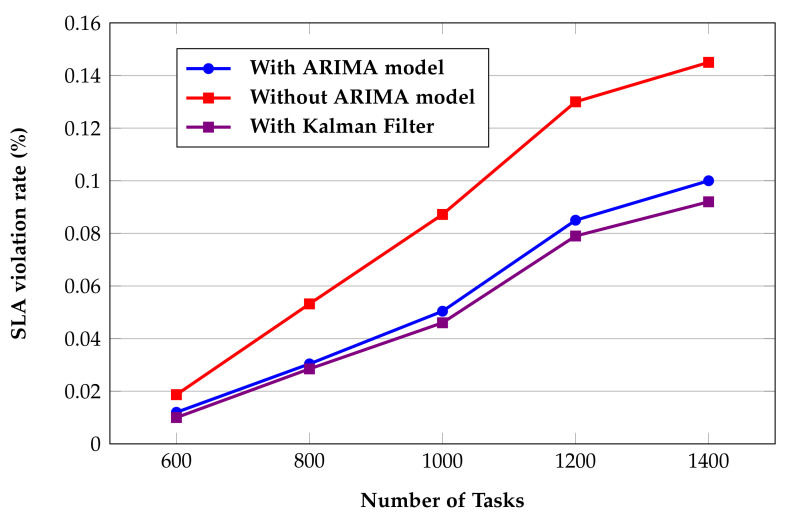
Effect of the prediction model on the proposed algorithm HPSO.

**Figure 11:**
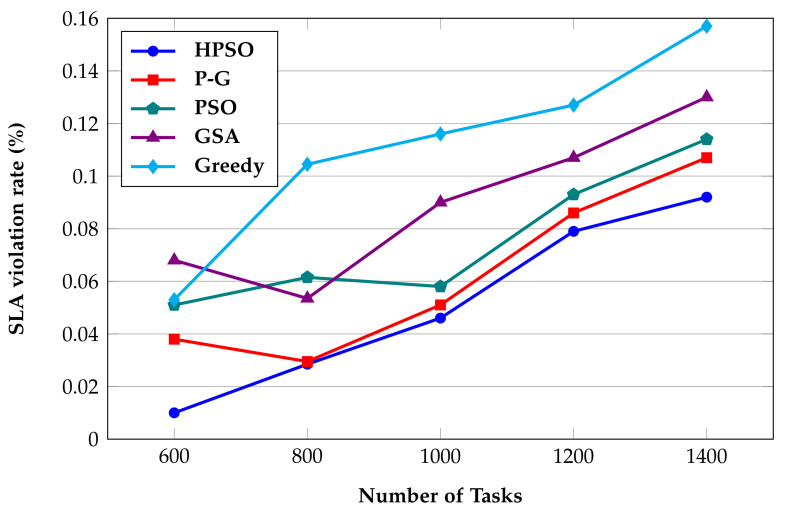
SLA violation rate comparison.

SLA violations occur when cloud service providers fail to provide services in accordance with SLA to users. The QoS guarantee in this paper, as part of the SLA, primarily ensures that the user’s tasks are completed on time, and does not include any further economic or legal guarantees. In this paper, the completion time of each task must not exceed a predefined deadline. Thus, SLA violation rate is defined as the percentage of tasks that are unable to be completed by their deadline. Figure 10 presents the effect of the prediction model proposed in this paper, and Figure 11 presents the comparison of the algorithms in term of SLA violation rate.

The values of *p*, *d*, and *q* in the ARIMA prediction model used in this paper are 2, 0, and 2, respectively [10]. The ARIMA model is optimized by using the Kalman filter JAVA package of the Efficient Java Matrix Library (EJML) (http://ejml.org/, accessed on 7 March 2022). It can be clearly seen from Figure 10 that the SLA violation rate is significantly better than without the prediction model. Since the ARIMA model is based on historical information and the current server status prediction, and due to the uncertainty of user task execution, it still cannot fully guarantee user QoS. The prediction model cannot achieve good results when physical resources are insufficient. It is found through experiments that when the number of tasks reaches 1400, the SLA violation rate using the prediction model is reduced by 0.045%. In addition, Figure 10 shows that the Kalman filter improved the ARIMA prediction model. Indeed, the SLA violation rate was reduced by 8% when using Kalman filter.

## 7. Conclusions

This paper is mainly based on the historical data and the monitoring information of the system to give an early warning to the user’s future QoS situation; it divides the user’s QoS early warning into three states and then uses a heuristic task flow scheduling algorithm to schedule tasks. The HPSO algorithm searches the solution space to find the most suitable solution for user QoS, and this solution should also minimize the cost consumption of the system. Finally, experiments show that our proposed HPSO algorithm reduces the execution cost by 16.51% compared to the algorithm of [11]. In addition, by using the ARIMA model optimized with the Kalman filter, the SLA violation rate is reduced to 0.092%, compared with the original 0.145%, when the number of tasks reaches 1400.

Several various adjustments, testing, and experiments remain to be completed in the future. Future work concerns exploring more objective functions and prediction improvements. Load balancing, makespan, and weighted completing time can be considered in the future as scheduling objectives. The ARIMA model is feasible as a prediction model, but the periodicity of user task data in the cloud computing data center and the user’s QoS preference attribute are not considered in the prediction model, so there is still a lot of room for improvement in the accuracy of prediction.

## Figures and Tables

**Table 1:** QoS early warning decision.

QoS Status	QoS Trends	Corresponding Decision
Normal area	QoS rises	Normal operation
	Slow degradation	Normal operation
	Medium-speed degradation	Normal operation
	High-speed degradation	Early warning
Warning area	QoS rises	Normal operation
	Slow degradation	Normal operation
	Medium-speed degradation	Early warning
	High-speed degradation	Early warning
Dangerous area	QoS rises	Normal operation
	Slow degradation	Early warning
	Medium-speed degradation	Early warning
	High-speed degradation	Early warning
Failure are	All	Alert

**Table 2:** Amazon EC2 virtual machine types.

Type	MIPS	RAM Size
Extra large instance	2500	850 MB
Large instance	2000	3750 MB
Medium instance	1000	1700 MB
Small instance	500	613 MB

## Data Availability

Not applicable.

## Abbreviations

The following abbreviations are used in this manuscript:

QoS	Quality of Service
ARIMA	Autoregressive Integrated Moving Average Model
HW	Holt–Winters
LSTM	Long Short-Term Memory
CRBM	Conditional Restricted Boltzmann Machine
CaaS	Computation-as-a-Service
AIMD	Additive Increase Multiplicative Decrease
SLA	Service-Level Agreement
PSO	Particle Swarm Optimization
GSA	Gravitational Search Algorithm
DAG	Directed Acyclic Graph
CPU	Central Processing Unit
AR	Auto Regressive
MA	Moving Average
ARMA	Autoregressive Moving Average Model
HPSO	Hybrid Particle Swarm Optimization
EC2	Elastic Compute Cloud
